# Supplementary material for: BOLD: Blood-gas and Oximetry Linked Dataset
Source: Sci Data. 2024 May 24;11:535. doi: 10.1038/s41597-024-03225-z (PMC11126612; doi:10.1038/s41597-024-03225-z)
Supplement: Supplementary file 1 — Supplementary Information file [file 41597_2024_3225_MOESM1_ESM.pdf]

Supplementary Information

Supplementary Information 1
Supplemental Figures 2
Supplemental Figure 1a. Completeness of the aligned covariates, among Asian patients 2
Supplemental Figure 1b. Completeness of the aligned covariates, among Black patients 2
Supplemental Figure 1c. Completeness of the aligned covariates, among Hispanic OR Latino patients 4
Supplemental Figure 1d. Completeness of the aligned covariates, among White patients 5
Supplemental Tables 6
Supplemental Table 1. Mapping of MIMIC-III, MIMIC-IV, eICU-CRD static concepts.. 6
Supplemental Table 2. Item IDs related to SaO2 and other laboratory test values, stratified by source database 7
Supplemental Table 3a. race and ethnicity unified mapping in MIMIC-IV 10
Supplemental Table 3b. race and ethnicity unified mapping in MIMIC-III 11
Supplemental Table 3b. race and ethnicity unified mapping in eICU-CRD 12
Supplemental Table 4. Descriptive patient characteristics by race and ethnicity 13
Supplemental Table 5. Descriptive patient characteristics by hidden hypoxemia (SpO2 ≥ 88% but SaO2 < 88%, as defined by Wong et al.2) 15

Supplemental Figures

Supplemental Figure 1a. Completeness of the aligned covariates, among Asian patients.

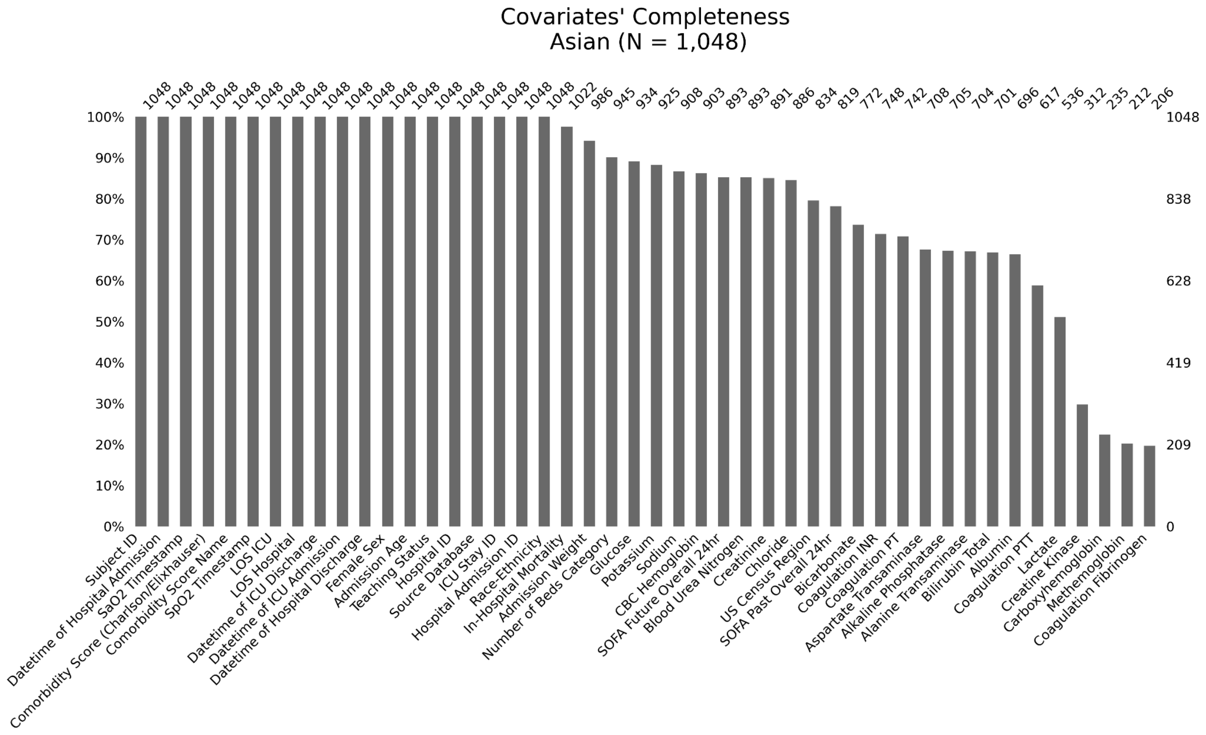

Supplemental Figure 1b. Completeness of the aligned covariates, among Black patients.

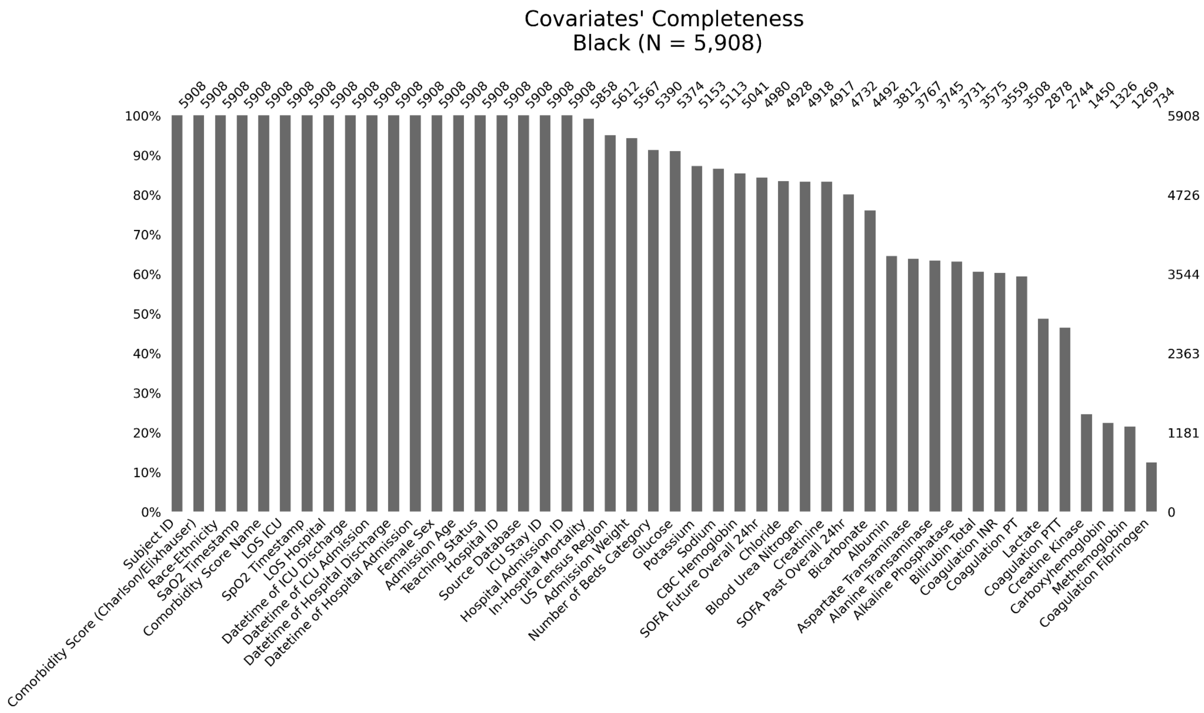

Supplemental Figure 1c. Completeness of the aligned covariates, among Hispanic OR Latino patients.

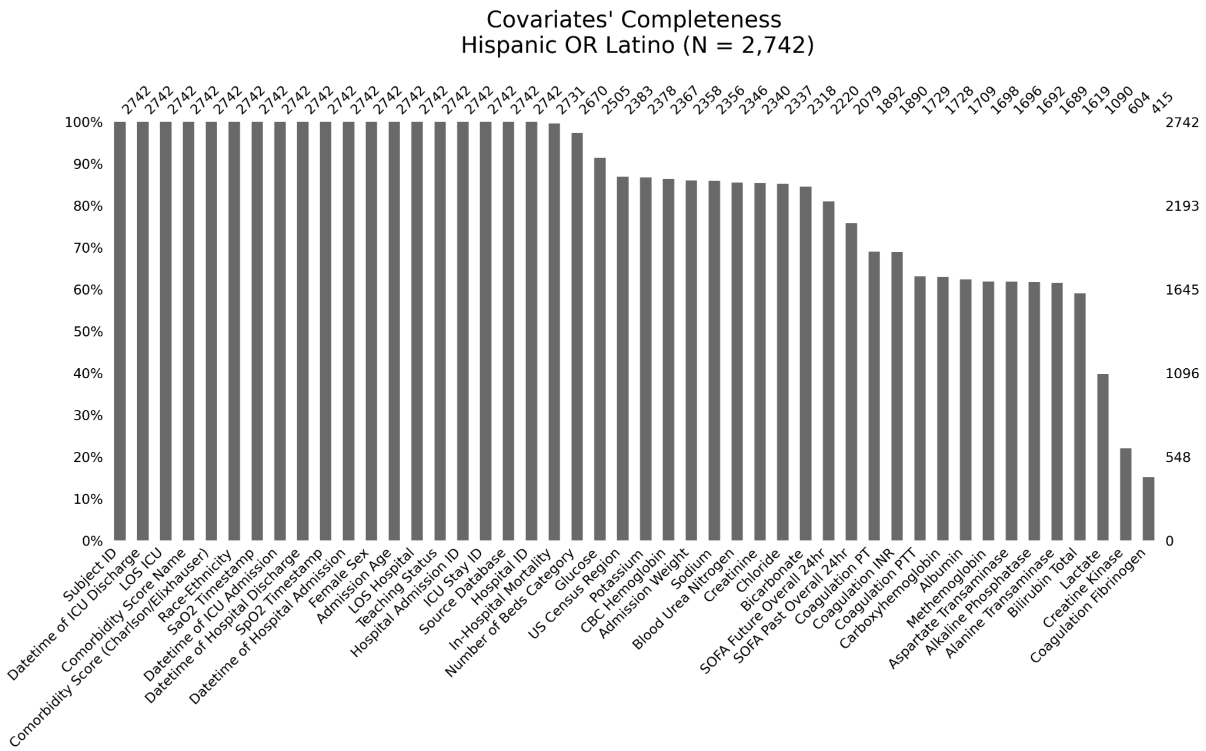

Supplemental Figure 1d. Completeness of the aligned covariates, among White patients.

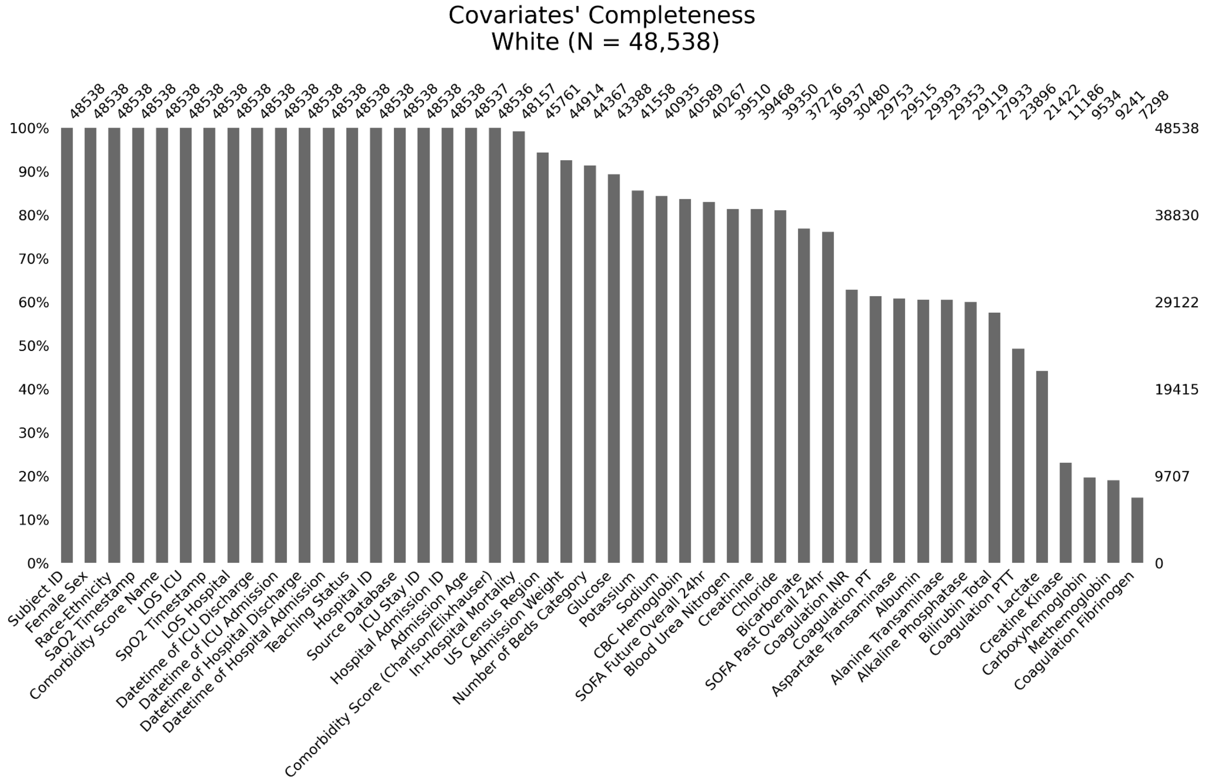

Supplemental Tables

Supplemental Table 1. Mapping of MIMIC-III, MIMIC-IV, eICU-CRD static concepts.

**Unified Concept**	**MIMIC-III**	**MIMIC-IV**	**eICU-CRD**
subject_id	SUBJECT_ID	subject_id	uniquepid
hospital_admission_id	HADM_ID	hadm_id	patienthealthsystemstayid
icustay_id	ICUSTAY_ID	stay_id	patientunitstayid
source_db	mimic_iii	mimic_iv	eicu
hospitalid	9999	9999	hospitalid
numbedscategory	≥ 500	≥ 500	numbedscategory
teachingstatus	TRUE	TRUE	teachingstatus
region	Northeast	Northeast	region
age (at admission)	> 90 = 90	> 90 = 90	> 89 = 90
sex_female	gender	gender	gender
weight_admission	weight_first	first_day_weight	admissionweight
height_admission	height_first	first_day_height	admissionheight
BMI_admission	weight_admission / ((height_admission / 100) ^ 2)		
datetime_hospital_admit	admittime	admittime	1 Jan 2014
datetime_hospital_discharge	dischtime	dischtime	1 Jan 2014 + hospitaldischargeoffset
datetime_icu_admit	intime	icu_intime	unitadmitoffset
datetime_icu_discharge	outtime	icu_outtime	unitdischargeoffset
los_hospital	los_hospital	los_hospital	hospitaldischargeoffset - hospitaladmissionoffset
los_ICU	los_icu	los_icu	icu_los_hours / 24
comorbidity_score_name	Elixhauser	Charlson	Charlson
in_hospital_mortality	hospital_expire_flag	hospital_expire_flag	hosp_mort

Supplemental Table 2. Item IDs related to SaO_2 and other laboratory test values, stratified by source database .

			itemid		label
**Type of Variable**	**Measurement**	**Left-side time window**	**MIMIC-III**	**MIMIC-IV**	**eICU-CRD**
**Arterial Blood Gas**	SaO_2	0	50817	50817	*O2 Sat (%)*
	paO_2	0	50821	50821	paO2
	$paCO_2$	0	50818	50818	paCO2
	pH	0	50820, 50831, 51094, 51491	50820	pH
	Methemoglobin	0	50814	50814	Methemoglobin
	Carboxyhemoglobin	0	50805	50805	Carboxyhemoglobin
**Vital Signs**	SpO_2	5min	646, 220277	220277	sao2
	Heart Rate	4h	211, 220045	220045	Heart Rate
	Respiratory Rate	4h	615, 618, 220210, 224690	220210, 224690	Respiratory Rate
	mbp_ni	4h	456, 443, 220181	220181	Non-Invasive BP Mean
	sbp_ni	4h	442, 455, 220179	220179	Non-Invasive BP Systolic
	dbp_ni	4h	8440, 8441, 220180	220180	Non-Invasive BP Diastolic
	mbp_i	4h	52, 6702, 220052, 225312	220052, 225312	Invasive BP Mean
	sbp_i	4h	51, 6701, 220050	220050, 225309	Invasive BP Systolic
	dbp_i	4h	8368, 8555, 220051	220051, 225310	Invasive BP Diastolic
	temperature	8h	223762, 676, 223761, 678	223761, 223762	Temperature (C)
**Complete Blood**	hemoglobin	24h	50811	51222	Hgb

**Count**					
	hematocrit	24h	50810	51221	Hct
	mch	24h	51248	51248	MCH
	mchc	24h	51249	51249	MCHC
	mcv	24h	51250	51250	MCV
	platelet	24h	51265	51265	platelets x 1000
	rbc	24h	51279	51279	RBC
	rdw	24h	51277	51277	RDW
	wbc	24h	51300	51301	WBC x 1000
**Coagulation**	fibrinogen	7 days	51214	51214	fibrinogen
	inr	7 days	51237	51237	PT - INR
	pt	7 days	51274	51274	PT
	ptt	7 days	51275	51275	PTT
**Basic Metabolic Panel**	sodium	24h	50983	50983	sodium
	potassium	24h	50971	50971	potassium
	chloride	24h	50806	50902	chloride
	bicarbonate	24h	50803	50882	bicarbonate
	bun	24h	51006	51006	BUN
	creatinine	24h	50912	50912	creatinine
	glucose	24h	50809	50931	bedside glucose, glucose
	aniongap	24h	50868	50868	aniongap
	calcium	24h	50808	50893	calcium
	lactate	24h	50813	50813	lactate
**Hepatic Function Panel**	alt	7 days	50861	50861	ALT (SGPT)
	alp	7 days	50863	50863	anion gap

	ast	7 days	50878	50878	AST (SGOT)
	bilirubin_total	7 days	50885	50885	total bilirubin
	bilirubin_direct	7 days	50883	50883	direct bilirubin
	albumin	7 days	50862	50862	albumin
**Enzyme**	ck_cpk	7 days	50910	50910	CPK
	ck_mb	7 days	50911	50911	CPK-MB
	ld_ldh	7 days	50954	50954	LDH

Supplemental Table 3a. race and ethnicity unified mapping in MIMIC-IV.

Original Labels in MIMIC-IV	N	Unified Mapping
AMERICAN INDIAN/ALASKA NATIVE	140	American Indian / Alaska Native
ASIAN	840	Asian
ASIAN - ASIAN INDIAN	179	Asian
ASIAN - CHINESE	792	Asian
ASIAN - KOREAN	51	Asian
ASIAN - SOUTH EAST ASIAN	293	Asian
BLACK/AFRICAN	309	Black
BLACK/AFRICAN AMERICAN	6723	Black
BLACK/CAPE VERDEAN	502	Black
BLACK/CARIBBEAN ISLAND	426	Black
HISPANIC OR LATINO	724	Hispanic OR Latino
HISPANIC/LATINO - CENTRAL AMERICAN	47	Hispanic OR Latino
HISPANIC/LATINO - COLUMBIAN	60	Hispanic OR Latino
HISPANIC/LATINO - CUBAN	72	Hispanic OR Latino
HISPANIC/LATINO - DOMINICAN	535	Hispanic OR Latino
HISPANIC/LATINO - GUATEMALAN	168	Hispanic OR Latino
HISPANIC/LATINO - HONDURAN	61	Hispanic OR Latino
HISPANIC/LATINO - MEXICAN	68	Hispanic OR Latino
HISPANIC/LATINO - PUERTO RICAN	902	Hispanic OR Latino
HISPANIC/LATINO - SALVADORAN	104	Hispanic OR Latino
MULTIPLE RACE/ETHNICITY	68	More Than One Race
NATIVE HAWAIIAN OR OTHER PACIFIC ISLANDER	110	Native Hawaiian / Pacific Islander
OTHER	2368	Unknown
PATIENT DECLINED TO ANSWER	425	Unknown
PORTUGUESE	322	White
SOUTH AMERICAN	64	Hispanic OR Latino
UNABLE TO OBTAIN	844	Unknown
UNKNOWN	6415	Unknown
WHITE	47197	White
WHITE - BRAZILIAN	147	White
WHITE - EASTERN EUROPEAN	164	White
WHITE - OTHER EUROPEAN	1307	White

WHITE - RUSSIAN	754	White

Supplemental Table 3b. race and ethnicity unified mapping in MIMIC-III.

**Original Labels in MIMIC-III**	**N**	**Unified Mapping**
AMERICAN INDIAN/ALASKA NATIVE	54	American Indian / Alaska Native
AMERICAN INDIAN/ALASKA NATIVE FEDERALLY RECOGNIZED TRIBE	3	American Indian / Alaska Native
ASIAN	1510	Asian
ASIAN - ASIAN INDIAN	93	Asian
ASIAN - CAMBODIAN	22	Asian
ASIAN - CHINESE	278	Asian
ASIAN - FILIPINO	27	Asian
ASIAN - JAPANESE	7	Asian
ASIAN - KOREAN	12	Asian
ASIAN - OTHER	18	Asian
ASIAN - THAI	4	Asian
ASIAN - VIETNAMESE	53	Asian
BLACK/AFRICAN	44	Black
BLACK/AFRICAN AMERICAN	5591	Black
BLACK/CAPE VERDEAN	206	Black
BLACK/HAITIAN	105	Black
CARIBBEAN ISLAND	9	Hispanic OR Latino
HISPANIC OR LATINO	1742	Hispanic OR Latino
HISPANIC/LATINO - CENTRAL AMERICAN (OTHER)	13	Hispanic OR Latino
HISPANIC/LATINO - COLOMBIAN	10	Hispanic OR Latino
HISPANIC/LATINO - CUBAN	24	Hispanic OR Latino
HISPANIC/LATINO - DOMINICAN	83	Hispanic OR Latino
HISPANIC/LATINO - GUATEMALAN	39	Hispanic OR Latino
HISPANIC/LATINO - HONDURAN	4	Hispanic OR Latino
HISPANIC/LATINO - MEXICAN	12	Hispanic OR Latino
HISPANIC/LATINO - PUERTO RICAN	237	Hispanic OR Latino
HISPANIC/LATINO - SALVADORAN	18	Hispanic OR Latino
MIDDLE EASTERN	44	White
MULTI RACE ETHNICITY	137	More Than One Race

NATIVE HAWAIIAN OR OTHER PACIFIC ISLANDER	18	Native Hawaiian / Pacific Islander
OTHER	1549	Unknown
PATIENT DECLINED TO ANSWER	567	Unknown
PORTUGUESE	70	White
SOUTH AMERICAN	9	Hispanic OR Latino
UNABLE TO OBTAIN	882	Unknown
UNKNOWN/NOT SPECIFIED	4724	Unknown
WHITE	42488	White
WHITE - BRAZILIAN	64	White
WHITE - EASTERN EUROPEAN	28	White
WHITE - OTHER EUROPEAN	85	White
WHITE - RUSSIAN	168	White

Supplemental Table 3b. race and ethnicity unified mapping in eICU-CRD.

**eICU-CRD**	**N**	**Unified Mapping**
–	2,290	Unknown
African American	21,308	Black
Asian	3,270	Asian
Caucasian	155,285	White
Hispanic	7,464	Hispanic OR Latino
Native American	1,700	American Indian / Alaska Native
Other/Unknown	9,542	Unknown

Supplemental Table 4. Descriptive patient characteristics by race and ethnicity.

		**Asian**	**Black**	**Hispanic OR Latino**	**White**
**N**	**Class**	858	4785	2116	37,380
**Covariates**					
Database	eICU-CRD	723 (84.3)	4405 (92.1)	1934 (91.4)	33,357 (89.2)
	MIMIC-III	16 (1.9)	42 (0.9)	20 (0.9)	590 (1.6)
	MIMIC-IV	119 (13.9)	338 (7.1)	162 (7.7)	3,433 (9.2)
Age (admission), median [Q1,Q3]		67.0 [55.0,77.0]	61.0 [50.0,71.0]	67.0 [53.0,78.0]	67.0 [57.0,77.0]
Sex,N (%)	Female	357 (41.6)	2290 (47.9)	965 (45.6)	16,217 (43.4)
BMI (Admission), median [Q1,Q3]		24.6 [21.8,28.4]	28.5 [23.8,35.0]	27.3 [23.7,32.1]	28.2 [23.9,33.6]
Charlson Comorbidity Index, median [Q1,Q3]		4.0 [2.0,6.0]	4.0 [2.0,6.0]	4.0 [2.0,6.0	4.0 [2.0,6.0]
Elixhauser Comorbidity Index, median [Q1,Q3]		12.0 [6.0,19.2]	13.0 [6.2,18.0]	7.0 [5.0,11.2]	8.0 [3.0,16.0]
SOFA past 24h, median [Q1,Q3]		4.0 [2.0,6.0]	4.0 [2.0,7.0]	5.0 [2.0,7.0]	4.0 [2.0,7.0]
Hospital Region, N (%)	Midwest	115 (17.3)	1205 (26.4)	148 (8.1)	11,646 (32.9)
	Northeast	169 (25.4)	487 (10.7)	230 (12.6)	7,073 (20.0)

	South	166 (25.0)	2469 (54.1)	1044 (57.4)	9,432 (26.7)
	West	215 (32.3)	404 (8.8)	397 (21.8)	7,199 (20.4)
**Outcomes**					
Hidden Hypoxemia, N (%)		22 (2.6)	216 (4.5)	70 (3.3)	1,314 (3.5)
ICU LoS if dead, median [Q1,Q3], days		4.7 [2.0,9.7]	4.2 [1.8,9.3]	3.5 [1.5,7.3]	3.4 [1.5,7.2]
ICU LoS if survived, median [Q1,Q3], days		3.2 [1.7,6.2]	3.3 [1.8,6.5]	2.8 [1.5,5.3]	3.0 [1.7,5.8]
In-Hospital Mortality, N (%)		686 (82.0)	3926 (82.7)	1712 (81.3)	6,484 (17.5)

Legend: LoS, length of stay

Supplemental Table 5. Descriptive patient characteristics by hidden hypoxemia ($SpO_2 \geq 88\%$ but $SaO_2 < 88\%$, as defined by Wong et al.[2]).

		Hidden Hypoxemia absent	Hidden Hypoxemia present
**N**	**Class**	47,362	1,731
**Covariates**			
Database	eICU-CRD	41,752 (88.2)	1,686 (97.4)
	MIMIC-III	722 (1.5)	18 (1.0)
	MIMIC-IV	4,888 (10.3)	27 (1.6)
Age (admission), median [Q1,Q3]		66.0 [55.0,76.0]	65.0 [54.0,75.0]
Race and Ethnicity, N (%)	American Indian / Alaska Native	371 (0.8)	9 (0.5)
	Asian	836 (1.8)	22 (1.3)
	Black	4,569 (9.6)	216 (12.5)
	Hispanic OR Latino	2,046 (4.3)	70 (4.0)
	Unknown	3,462 (7.3)	100 (5.8)
	White	36,066 (76.1)	1,314 (75.9)
	More Than One Race	3 (0.0)	
	Native Hawaiian / Pacific Islander	9 (0.0)	
Sex, N (%)	Female	20,634 (43.6)	799 (46.2)
BMI (Admission), median [Q1,Q3]		28.1 [23.8,33.5]	28.2 [23.8,35.6]
Charlson Comorbidity Index, median [Q1,Q3]		4.0 [2.0,6.0]	4.0 [2.0,6.0]

Elixhauser Comorbidity Index, median [Q1,Q3]		9.0 [3.0,16.0]	14.0 [7.2,18.5]
SOFA over past 24 hours, median [Q1,Q3]		4.0 [2.0,7.0]	4.0 [2.0,7.0]
Hospital Region, N (%)	Midwest	13,334 (30.0)	645 (39.0)
	Northeast	8,802 (19.8)	250 (15.1)
	South	13,564 (30.5)	454 (27.4)
	West	8,764 (19.7)	305 (18.4)
**Outcomes**			
ICU LoS if dead, median [Q1,Q3], days		3.6 [1.6,7.7]	2.6 [1.0,5.9]
LoS ICU if survived, median [Q1,Q3], days		3.0 [1.7,5.9]	3.6 [1.9,7.0]
In-Hospital Mortality, N (%)		8,092 (17.2)	450 (26.1)
